# Bio-inspired special wettability in oral antibacterial applications

**DOI:** 10.3389/fbioe.2022.1001616

**Published:** 2022-08-30

**Authors:** Xin Zhang, Rushui Bai, Qiannan Sun, Zimeng Zhuang, Yunfan Zhang, Si Chen, Bing Han

**Affiliations:** ^1^ Department of Orthodontics, School and Hospital of Stomatology, Peking University, Beijing, China; ^2^ National Engineering Laboratory for Digital and Material Technology of Stomatology & Beijing Key Laboratory of Digital Stomatology, Beijing, China

**Keywords:** bio-inspired, super wettability, low-fouling surfaces, antibacterial, oral biofilm management

## Abstract

Most oral diseases originate from biofilms whose formation is originated from the adhesion of salivary proteins and pioneer bacteria. Therefore, antimicrobial materials are mainly based on bactericidal methods, most of which have drug resistance and toxicity. Natural antifouling surfaces inspire new antibacterial strategies. The super wettable surfaces of lotus leaves and fish scales prompt design of biomimetic oral materials covered or mixed with super wettable materials to prevent adhesion. Bioinspired slippery surfaces come from pitcher plants, whose porous surfaces are infiltrated with lubricating liquid to form superhydrophobic surfaces to reduce the contact with liquids. It is believed that these new methods could provide promising directions for oral antimicrobial practice, improving antimicrobial efficacy.

## Introduction

**FIGURE 1:**
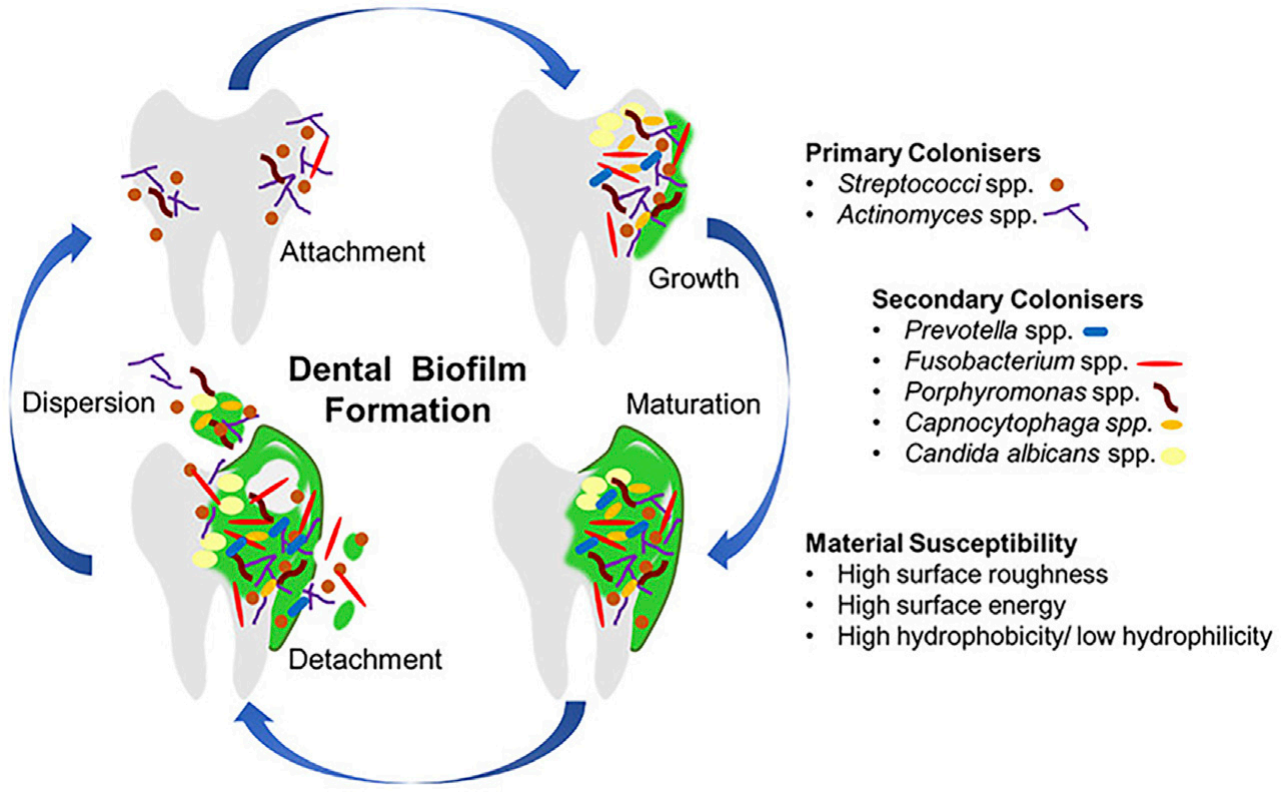
The progress of dental biofilm formation. Copyright from ref (Ramburrun et al., 2021).

Most oral diseases originate from plaque biofilms, different compositions and locations of which result in distinct diseases. Acid-producing plaques on the edges of teeth, orthodontic archwires or brackets, or restorations lead to caries; residual biofilm after root canal treatment may lead to inflammation recurrence and even apical paracentesis; subgingival plaque of teeth and implants may lead to periodontitis and periimplantitis; biofilms on dentures are associated with local and general inflammation, such as mucosal inflammation and aspiration pneumonia (Li et al., 2010; Jiao et al., 2019; Park et al., 2020). As shown in Figure 1(Ramburrun et al., 2021), biofilm forms as follows: bacteria attachment, growth, maturation, and dispersion (Jiao et al., 2019). A glycoprotein is naturally present in saliva, which makes almost all surfaces in the mouth being covered with it, and bacteria can adhere to it (Ramburrun et al., 2021). In the process of plaque biofilm formation, first bacteria attaching to teeth are called pioneer species, such as *Streptococcus spp.* and *Actinomyces spp*. These pioneer species promote the subsequent colonization and create anoxic conditions, which play a crucial role in the formation and maturation of biofilm (Lad et al., 2014). Above progresses suggest that inhibiting the adhesion of pioneer bacteria is the premise of suppressing palque formation.

Major materials used for inhibiting biofilm formation rely on bactericidal effect, including antibiotics, chlorhexidine, fluoride, etc. However, drug resistance limits the use of antibiotics; chlorhexidine has a limited effect; antibacterial effect shows in a high concentration of fluoride, that is, difficult to achieve and accompanied by some toxicity (Autio-Gold, 2008; Rošin-Grget et al., 2013; Oh et al., 2017; Ullah et al., 2017; Zhou et al., 2021). Current limitations call for new antibacterial materials. Metal ions and polymetric antimicrobial materials are developed to eliminate bacteria but also face problems such as the toxicity of metal ions (Li et al., 2021; Ramburrun et al., 2021). Zhou et al. used programmable base pair interactions at the nanoscale to make a encapsulated quaternary ammonium group within the dense hydroxyapatite that endowed the composite with long-lasting and local antibacterial activity (Zhou et al., 2022). Now that attachment of salivary proteins and pioneer microbial species is the first step in plaque formation and intermediated by water (Lendenmann et al., 2000; Donlan and Costerton, 2002), managing surface wettability might be a promising and simple solution to control this process.

**FIGURE 2:**
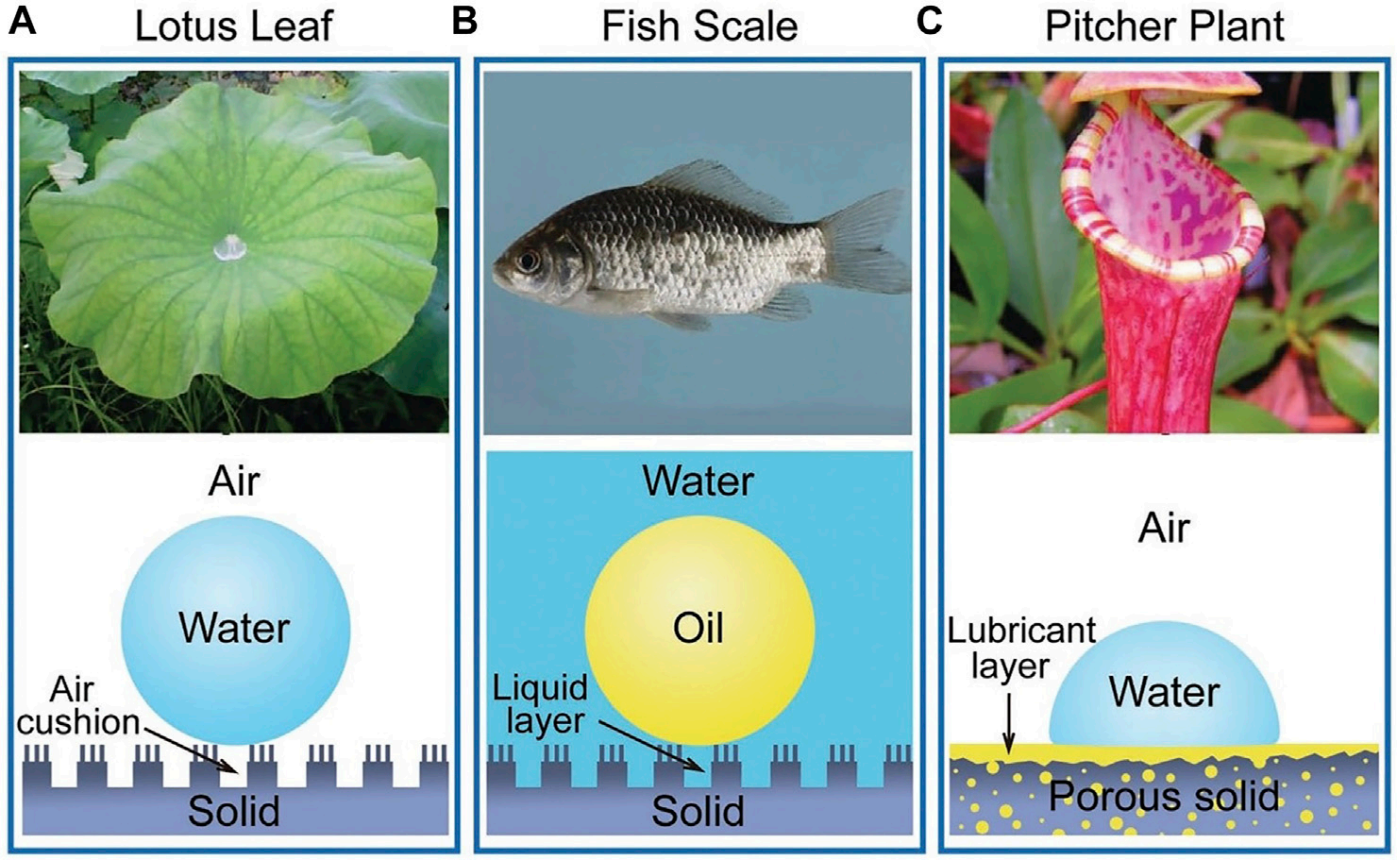
The natural anti-fouling surfaces. Copyright from ref (Cao et al., 2016).

Many biological antifouling phenomena in nature are based on special wettability. For example, lotus leaf is superhydrophobic surface to trap a stable air cushion so that outside water has less access to the surface, achieving antifouling (Figure 2A); fish scales can trap a water layer through the super hydrophilic surface to reduce the adhesion of oil in water (Figure 2B); picher plant uses the porous surfaces infiltrating with lubricating liquid to reduce contact with other liquid (Figure 2C) (Cao et al., 2016). The wettability of surface is expressed by the contact angle (CA) of a water droplet on the substrate. Young’s equation of CA is

$\cos\theta_{Y}=\frac{\gamma SV-\gamma SL}{\gamma LV}.\gamma SV,\gamma SL,$ and γLV are the solid-liquid, solid-vapor, and liquid-vapor interfacial energies respectively. The CA of hydrophobic surface is >90°; the CA of hydrophilic surfaces is <90°. Proteins and bacteria tend to adhere to a slightly hydrophobic or hydrophilic surface, while less adhesion occurs on the highly hydrophobic or hydrophilic surfaces. Therefore, anti-fouling effect could be achieved by changing wettability of teeth or material surfaces via increasing hydrophobicity (e.g., silica-based materials), like lotus leaves, or hydrophilicity (e.g., zwitterion), like fish scales; or via inspired slippery liquid-infused porous surfaces. In this review, we will summarize the principles and synthesis of bio-inspired materials with super wettability to prevent adhesion, and focus on their dental applications (Table 1.).

**TABLE 1:** The summary of super wettable material applied in dentistry.

Material	Wettability	Character	Disadvantage
Poly ethylene glycol	Super hydrophilicity	• Form a water layer on the surface. The layer can reduce adhesion	• Poor stabilization
		• A biocompatible polymer	• Lose the anti-fouling ability at 35°
		• Be grafted to substrate or coupled to polyelectrolytes directly	
		• Use silane chemistry to combine with orthodontic wires	• Not be metabolized naturally
		• Use free radical polymerization to synthesize polymers that be grafted to resin	
Zwitterionic polymers (2-methacryloyloxyethyl phosphorylcholine, sulfobetaine methacrylate)	Super hydrophilicity	• Lock a layer of free water on the surface to form a physical or energetic barrier	• May degrade the mechanical properties of mixed materials
		• Coat teeth or enamel directly	
		• Reduce the “coat-inhibition” of other bactericidal materials	
		• Physically mix with flowable resin, inorganic salt materials et al	
		• Graft to or from metal	
Proteins (histatin 5, casein phosphor peptide, bovine serum albumin)	Super hydrophilicity	• Anti-adhesion and promote remineralization	• Need more *in vitro* studies
		• Coat teeth or enamel	
		• Coat orthodontic archwires	
Silica-based materials	Super hydrophobicity	• Supper hydrophobicity reduces the temporal window and spatial possibilities for bio-adhesion events of bacteria from a contaminated droplet.	• The anti-fouling of surfaced created by chemical modification may be not durable
		• Coat titanium implant and orthodontic archwires	
		• Introduced into the resin by branched amino silicone	
Slippery liquid-infused porous surfaces (SLIPS)	Super hydrophobicity (bioinspired slippery surfaces)	• Low-surface-energy porous solids are infiltrated by lubricating liquids to form a stable, immobilized, and smooth liquid-like omniphobic surface	• SLIPS has a little application
		• Immiscible liquids deposited on the SLIPS can be easily removed even under weak shear forces	• Need more *in vivo* and *in vitro* experiments

## Super-hydrophilic material

The surface of fish scales is covered with a layer of hydrophilic components, as well as special nano-structures, which trap water on the surface underwater to resist oil (Liu et al., 2009). Similarly, the teeth or oral material surfaces could also be modified to be super hydrophilic to form a hydration layer. The tightly bound water layer forms a physical or energetic barrier, making it difficult for microorganisms to adhere and penetrate (Chen et al., 2010; Cazzaniga et al., 2015; Leng et al., 2016).

### Poly ethylene glycol

Poly ethylene glycol (PEG) is a biocompatible polymer. PEG has pretty hydrophilicity and can reduce the adhesion of proteins, platelets, and bacteria (Elbert and Hubbell, 1998; Park et al., 1998; Chen et al., 2000; Kenausis et al., 2000; Razatos et al., 2000; Zhu et al., 2001; Harris, 2013). Then anti-adhesion is due to the strong hydrophilicity of polyethylene glycol, which forms a water layer on the surface. The layer can reduce the adsorption of proteins (Harder et al., 1998; Feldman et al., 1999; Harris, 2013). PEG can connect with different terminal functional groups to reduce the protein adhesion to different extents. A relatively long PEG surface will have better resistance (Park et al., 1998).

**FIGURE 3:**
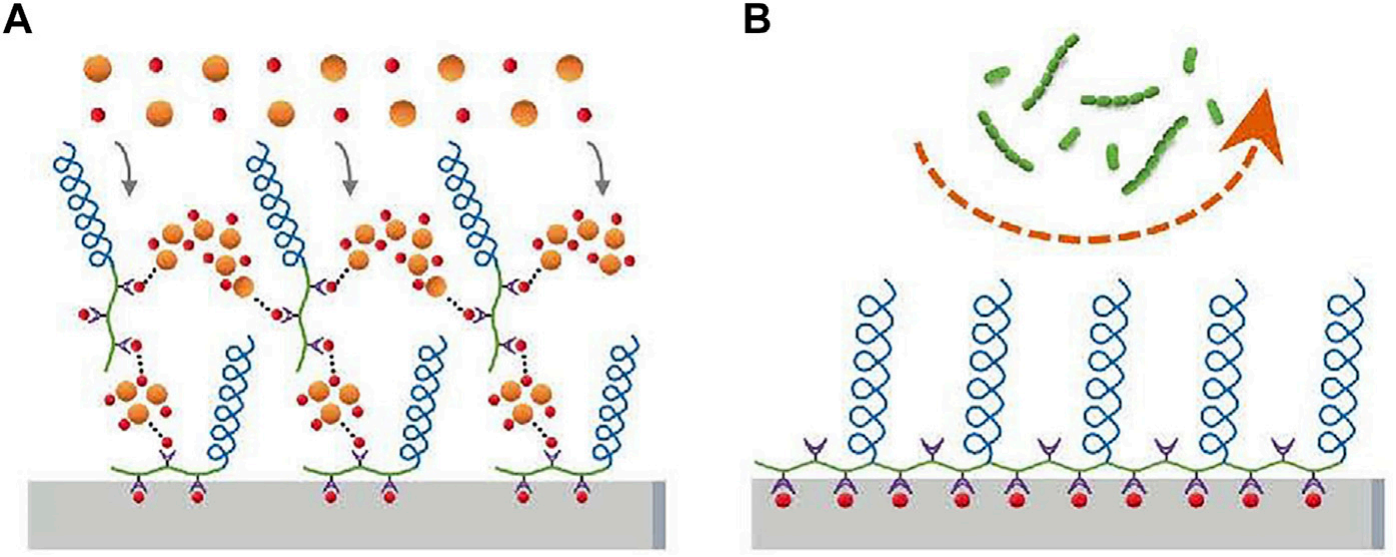
PASP-PEG on the enamel surface forms a brush-like barrier that inhibits bacterial adhesion (*S. sanguis* and *S. mutans*). Copyright from ref (Hou et al., 2020).

PEG can be grafted to substrate or coupled to polyelectrolytes directly, such as poly (l-lysine) (PLL) or poly (acrylic acid), and then adhere to the substance as a monolayer (Boulmedais et al., 2004). The layer-by-layer self-assembly of polyelectrolytes on charged surfaces offers another possibility to deposit polyelectrolytes with grafted PEG onto substrates (Decher and Hong, 1991). PEG can be used to compound a comb-like graft copolymer (poly (l-lysine)-grafted-poly (ethylene glycol)—PLL-g-PEG), with a polycationic PLL backbone and PEG side chains. The polymers can be adsorbed to negatively charged metallic oxide spontaneously, such as titanium or niobium oxide surfaces, to form a stable, densely packed PEG monomolecular adlayer to reduce the adhesion of bacteria (Harris et al., 2004). Poly (aspartic acid)-polyethylene glycol (PASP-PEG) was synthesized by a similar method with high affinity for hydroxyapatite (HA)/tooth surfaces and low toxicity, and promoted mineralization of PASP, like mineralization protein (Hou et al., 2020). And the reason for antiadhesion is as follows (Figure 3): Firstly, a water layer is formed due to hydrophilicity (Lüsse and Arnold, 1996; Aray et al., 2004); in addition, “steric repulsion” can be obtained from the long chain of PASP-PEG, which is an entropic effect concerning the change in free energy associated with confinement and the dehydration of soft polymer chains (Hui et al., 2017). PEG can not only be coupled to polyelectrolytes but also be inserted into polyelectrolyte multilayer. It has been reached that poly (l-glutamic acid)-grafted-poly (ethylene glycol) (PGA-g-PEG) is obtained by modifying the PGA backbone by a PEG, which is inserted into polyelectrolyte multilayer to get the same anti-adhesive effect (Boulmedais et al., 2004). Cui (Cui et al., 2016) used conventional free radical polymerization and changed the feed ration of monomers to synthesize a series of copolymers containing pendants of poly (ethylene glycol) methyl ether methacrylate (PEGMA) and ethylene glycol methacrylate phosphate (Phosmer). And then the copolymer was anchored to hydroxyapatite and enamel to provide inhibition of bacterial adhesion.

**FIGURE 4:**
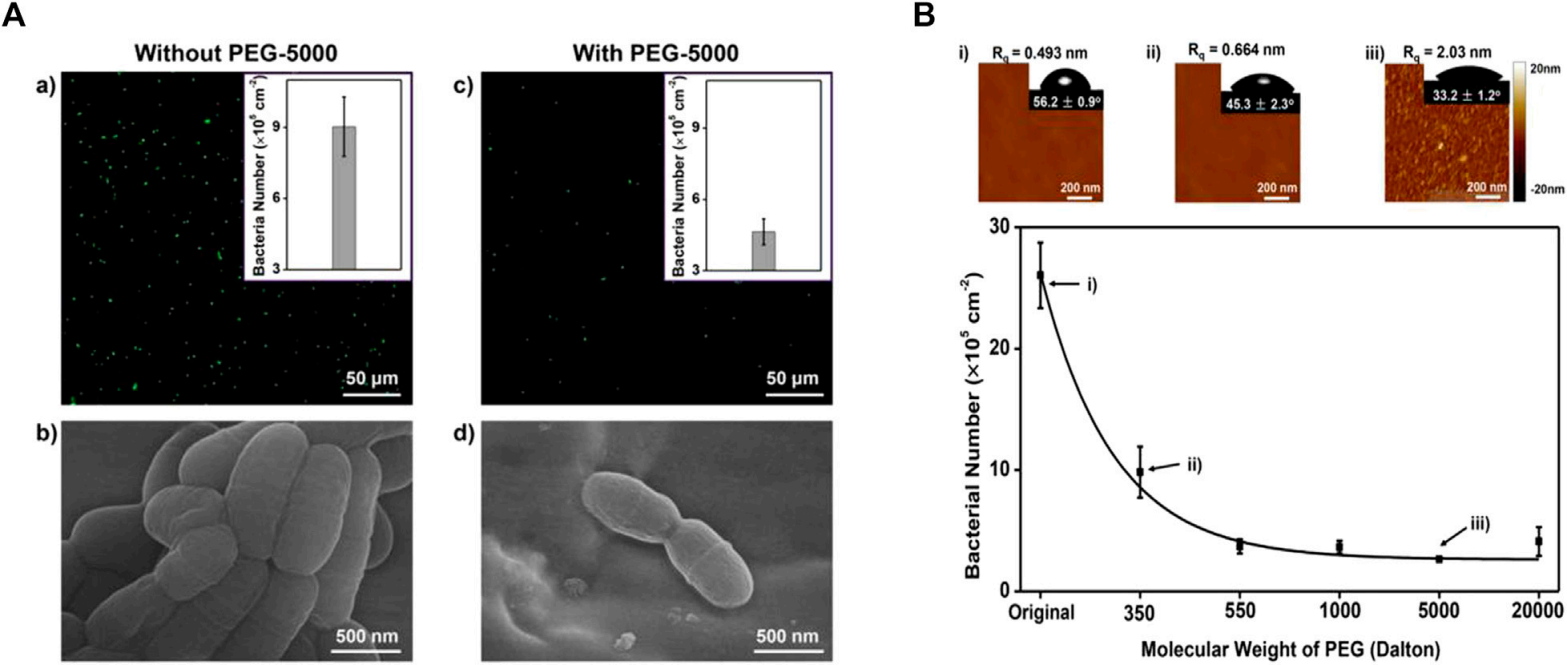
**(A-a,b)** For the stainless steel archwires without PEG coating, significant *S. mutans* adhesion was observed. **(A-c,d)** For the stainless steel archwires coated with PEG of molecular weight 5,000 (PEG-5000), the number of adhered *S. mutans* was greatly reduced. **(B)**When the molecular weight of PEG was increased from 350 to 20,000, the anti-adhesive property of PEG-coated stainless steel archwires increased, which may be due to the relative hydrophilicity of long-chain PEG-modified stainless steel archwires. Reprinted (adapted) with permission from (Peng et al., 2017). Copyright 2017 American Chemical Society.

Besides coating, PEG hydrogel has been applied in the field of wound dressing, drug delivery, etc (Vimala et al., 2010; Li et al., 2011; Dong et al., 2016; Nitta et al., 2017; Wang et al., 2017; Zhao et al., 2017; Bozuyuk et al., 2018; Shutava et al., 2019). Peng et al. (Peng et al., 2017) bond long-chain PEG chemically and used silane chemistry to combine it with orthodontic wires to avoid *S. mutans* adhesion. It has been demonstrated that PEG can use hydrogen bonding to form a stable water layer to resist adhesion (Figure 4). In their next study (Peng et al., 2020), they used chitosan (CS) and PEG to synthesize a hydrogel by silanization and copolymerization reaction, covering the stainless steel wire. The hydrogen consists of cross-linked PEG and CS chains. The cross-linked PEG can absorb water effectively through hydrogen bonds to form a thin water layer to provide a pretty anti-adhesive performance, while CS can provide bactericidal function. The antibacterial performance is best when CS/PEG. The surface charge becomes more positive when the portion of CS increased and the anti-adhesive performance will be better.

PEG can also be used in resin-based composites. Poly (ethylene glycol) methyl ether methacrylate (PEGMA) can be synthesized by PEG through free radical polymerization and has been certified to resist biological contamination (Tedjo et al., 2007; Cui et al., 2016). PEGMA can be grafted to polymethyl methacrylate (PMMA) by atmospheric pressure plasma to improve the hydrophilic and anti-adhesive properties of PMMA. It can prevent bacterial adhesion effectively, even though it is coated with salivary. However, this method exposes the alkoxy portion of the glycol chain, so that the degree of hydrophilic improvement is limited, for the hydrophilicity of the alkoxy portion is less than that of hydroxyl (Cui et al., 2016; Lee et al., 2018).

Though PEG was approved to be used in humans in 1992, and the prospects for its application are greatly enhanced, there are still many problems. The stabilization of PEG is poor. It will autoxidize and degrade during storage or handling at room temperature, especially in the presence of transition metal ions, which are present in most biological solutions (Hamburger et al., 1975; Crouzet et al., 1976; Gerhardt and Martens, 1985). Studies have shown that when the temperature rises to 35 °C, PEG brushes lose the anti-fouling ability (Leckband et al., 1999). Moreover, PEG can not be metabolized naturally. These limitations should be considered when applying PEG to oral materials.

### Zwitterionic polymers

As for the disadvantages of PEG, the zwitterionic polymer has been considered to be the perfect alternative. Compared with the amphiphilicity of PEG, zwitterionic polymers are super hydrophilic due to the presence of abundant ions and subsequent strong hydration layers (Zheng et al., 2017). The main zwitterions used in dentistry are 2-methacryloyloxyethyl phosphorylcholine (MPC) and sulfobetaine methacrylate (SBMA), of which MPC is the most common.

**FIGURE 5:**
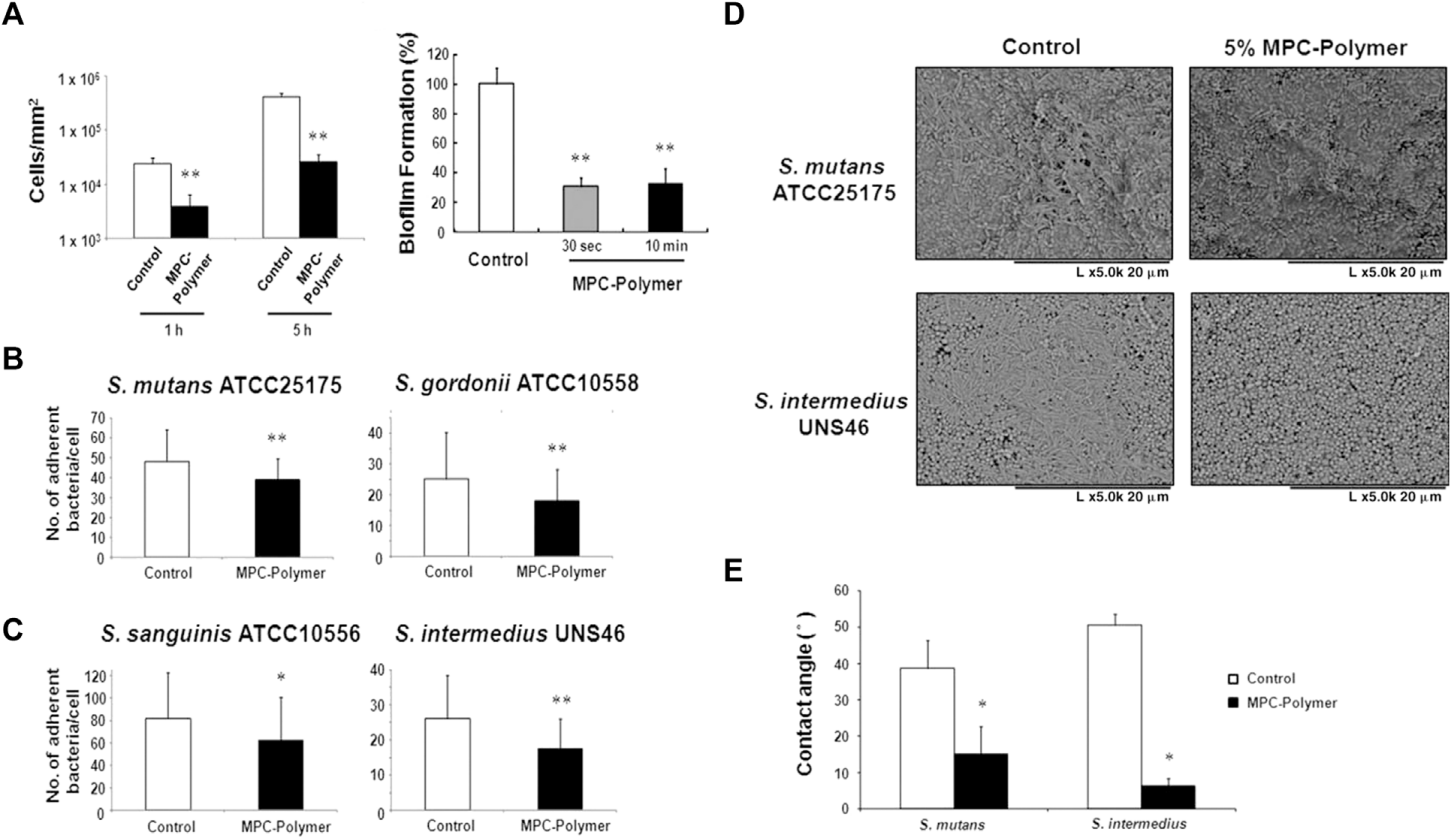
**(A–C)**The effects of MPC-polymer treatment on streptococcal adherence to saliva-coated hydroxyapatite and oral epithelial cells, and biofilm formation of *S. mutans* on saliva-coated hydroxyapatite. The mean number of adherent bacteria ±SD to 1 cell was calculated. *p < 0.05 and **p < 0.01 compared with the control (without MPC-polymer treatment). The results are representative of 5 different experiments demonstrating similar results. **(D, E)** The effect of MPC-polymer on the adherence of *F. nucleatum* to saliva-coated streptococcal biofilms. MPC-polymer treatment significantly inhibited the adherence of *F. nucleatum* JCM8532 to both S. mutans ATCC25175 and *S. intermedius* UNS46 biofilms when compared with the non-treated control. **(D)** After cultivation, the adhesion of *F. nucleatum* to the streptococcal biofilm was observed by SEM. The results are representative of 5 different experiments demonstrating similar results. **(E)** As an index of hydrophobicity, the surface contact angles of streptococcal biofilm were measured by the horizontal projection technique. *p < 0.01 compared with the control (without MPC-polymer treatment). The results are representative of 5 different experiments demonstrating similar results. Copyright from ref (Hirota et al., 2011).

MPC is a methacrylate with a phospholipid polar group in the side chain (Lewis, 2000). The phospholipids, as the main components of the cell membrane, consist of a hydrophilic head and a hydrophobic tail, so they can form lipid bilayers that have the hydrophilic head to the outside and the hydrophobic tail to the inside, which contribute to the super hydrophilicity of MPC (Ishihara et al., 1990; Mashaghi et al., 2013). Due to the super hydrophilicity, MPC can lock a layer of free water on the surface, which can effectively detach proteins to reduce the adsorption (Ishihara et al., 1998; Yamasaki et al., 2003). On the other hand, the water layer can form a physical or energetic barrier, making it difficult for microorganisms to penetrate or adhere (Chen et al., 2010; Cazzaniga et al., 2015; Leng et al., 2016). K. Hirota’s group (Hirota et al., 2011) first demonstrated that MPC polymers significantly inhibit the adhesion of many oral bacteria to hydroxyapatite and oral epithelial cells *in vitro*, therefore effectively reducing plaque formation (Figure5). And then it has been demonstrated that MPC can be merged with 2-methacryloyloxyethyl phosphate (MOEP) monomers. For MOEP has Ca^2+^-binding moieties that can be combined with hydroxyapatite, the compound makes MPC bond with teeth directly, forming a pretty anti-biofouling coating (Kang et al., 2016). Besides teeth, Yumoto (Yumoto et al., 2015) showed that the interaction of butyl in MPC with hydrophobic structural domains in surface proteins of oral epithelial cells made MPC adhere to the oral epithelium and the hydrophilicity of MPC prevented *Porphyromonas gingivalis* from adhering the epithelium. In addition, while MPC adhered to the epithelium, it could prevent periodontics by blocking the binding of TLR2 to reduce producing IL-8 and the natural immune mediated by IL-8. Recent clinical trials using mouthwash containing MPC showed that MPC didinhibit the increase of oral bacteria, especially *Streptococcus pyogenes* (Fujiwara et al., 2019).

**FIGURE 6:**
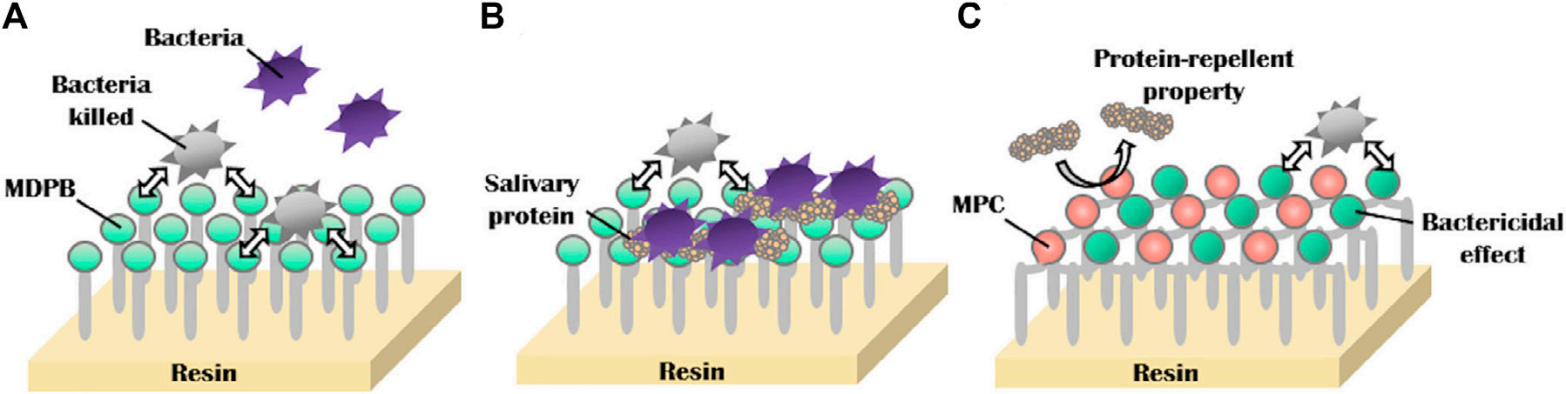
Schematic of dual functions of protein repellent property and antibacterial effect. **(A)** Dental resins with immobilized bactericides exhibit antibacterial effects, which depend on the contact inhibition of bacteria. **(B)** However, their effectiveness can be readily reduced by coverage with salivary protein. **(C)** Novel surface coating composed of 12-methacryloyloxydodecylpyrimidinium bromide and 2-methacryloyloxyethyl phosphorylcholine exhibits protein repellent ability and bactericidal effect (Thongthai et al., 2020). Copyright from ref (Thongthai et al., 2020).

MPC can be applied not only todirect anti-adhesion, but also to provide synergistic effects in combination with other antimicrobial agents. Many current oral anti-bacterial materials face the problem that direct contact is required for these materials toinhibit bacteria. In other words, this “contact-inhibition” effect is reduced if the surfaces are covered with salivary proteins. Methacryloyloxydodecylpyridinium bromide (MDPB) is a compound of the antibacterial agent dodecyl pyridinium bromide and a methacryloyl group, and it can copolymerize with other dental monomers (Imazato et al., 1995; Imazato, 2003). MDPB has an obvious limitation of contact inhibition, and introducing MPC into it can reduce the adsorption of salivary, which is conducive to the direct release of antibacterial components to achieve a better effect (Figure 6) (Thongthai et al., 2020). Therefore, combining hydrophilic MPC with hydrophobic dental restorative materials has become the main direction of current research. Hatsuno, Ishihara, and Nishigochi et al. found that water-soluble MPC could be combined with n-butyl methacrylate (BMA) to form an insoluble copolymer coating, which could be coated on the surface of resinous materials via hydrophobic interaction between the hydrophobic unit of BMA and resin. Pasiree Thongthai used a similar way to introduce MPC into a copolymer that was synthesized by radical polymerization of MDPB, MPC, and BMA in ethanol using 2,20azobisisobutyronitrile (AIBN) as an initiator, and MDPB, MPC, and BMA at mole ratios of 15:15:70. MPC reduced protein adhesion significantly and synergized with MDPB to enhance antibacterial ability (Thongthai et al., 2020). Referring to the experience of MDPB, MPC was subsequently combined with other antimicrobial materials, such as QAM, to overcome the problem of contact-inhibition of antimicrobial materials (Zhang et al., 2015b).

According to the above, MPC can also be combined with other materials in dentistry, e.g., with mineralization-promoting adhesives to provide antibacterial, anti-adhesive, and remineralization-promoting effects (Xie et al., 2017). MPC can be introduced into 3D printing material- PMMA - to achieve anti-adhesive 3D printing without affecting mechanical properties and printing accuracy (Kwon et al., 2021). In addition, MPC can be mixed with a variety of inorganic salt materials in the form of handmade powders to enhance the resistance of bacterial adhesion, such as light-curing fluorine coatings, root canal therapy material, and surface pre-reacted glass-ionomer (Kwon et al., 2019a; Kwon et al., 2019b; Lee et al., 2019). MPC can also be physically mixed and stirred with flowable resin, which also gives the orthodontic bonding agent an antibacterial effect (Park et al., 2020).

MPC has been approved by FDA for its good biocompatibility. But the mechanical properties of dental materials with MPC are degraded, especially when the MPC content is higher than 3% (Zhang et al., 2015a; Kwon et al., 2021).

SBMA and MPC are both amphoteric ions with similar anti-adhesive principles (Zheng et al., 2017). SBMA can be added to PMMA to achieve anti-adhesion (Kwon et al., 2021). SBMA can also be combined with metal, such as titanium and stainless steel, by methods of grafting “grafting from” or “grafting to” (Chou et al., 2017). “Grafting from” methods consist of growing a polymer from a monomer mixture at the surface of the material to modify, and bond it covalently to the surface. The “grafting to (or onto)” methods consist of bonding a polymer at the surface of the material at play and an efficient method to graft zwitterionic heads by this technique is to use glycidyl methacrylate, a biomimetic anchoring group (Zanini et al., 2007; Li et al., 2008; Gao et al., 2009; Xu et al., 2009; Huang et al., 2012; Schlenoff, 2014; Chou et al., 2016).

### Peptide

Faced with the above limitations of polyethylene glycols and amphoteric compounds, new strategies have been proposed: protein.

Histatin 5(H5) is a salivary antimicrobial peptide (AMP), that is, naturally present in the salivary glands and is very effective in killing bacteria including *S. mutans* (Madhwani and McBain, 2012; Krzyściak et al., 2015). What’s more, it can adhere to enamel well and inhibits demineralization (Yin et al., 2003; Siqueira et al., 2010). Compared with PEG and zwitterion, it has better biocompatibility (Zhou et al., 2021). AMP performance can be enhanced by grafting phosphoserine (S*p*)—a key component in initiating free calcium ion mineralization—to the N-terminal of H5 (Zhou et al., 2020). Later, Zhou et al. continued to set the end of S*p*S*p* (DSP) to increase the S*p* structure, which can enable enamel bound with modified H5 to have a pretty hydrophilicity and to resist bacterial adhesion through forming a thin layer of water on the surface (Zhou et al., 2021).

For enhancing the remineralization of teeth, another protein has been found and it can also be anti-adhesive. Casein phosphor peptide (CPP) is a natural phosphorylated peptide in milk that can be obtained by the proteolysis of casein (Meisel et al., 2003; Baum et al., 2013; Dallas et al., 2016). It can bind calcium and improve the remineralization of teeth effectively (Reynolds, 1987; Nongonierma and FitzGerald, 2012). It has been studied that CPP is a negatively charged amphiphilic polypeptide with the hydrophilic end facing outward, which can inhibit the initial adhesion of the *S. mutans* to hydroxyapatite by increasing the hydrophobicity of the HA surface and negative charge (Reynolds and Wong, 1983; Roger et al., 1994; Schüpbach et al., 1996; Fitzgerald, 1998; Colloca et al., 2000; Cross et al., 2005; Song et al., 2015; Yang et al., 2017; Wang et al., 2020).

As for the shortcomings of PEG that it tends to auto-oxidize into aldehydes in the presence of oxygen (Hucknall et al., 2009), Liu *et al.* actively explored other proteins to improve the anti-adhesive effect of orthodontic archwires. Bovine serum albumin (BSA) is an inexpensive and easily available protein with potent anti-adhesive properties to mammalian cells, platelets, and red blood cells (Cai et al., 2015; Jeoung et al., 2015). So Liu et al. chose BSA to be grafted onto orthodontic brackets, resisting the adhesion of bacteria (Liu et al., 2018).

## Super-hydrophobic material

**FIGURE 7:**
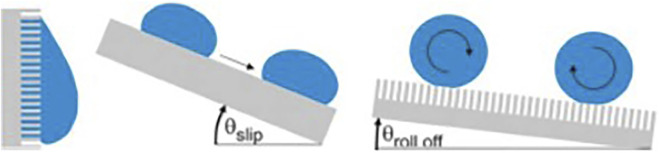
Water droplets on super-hydrophobic surfaces. Copyright from (Sterzenbach et al., 2020).

Hydrophobic surface of lotus leaf has high CA, giving it anti-fouling ability. This characteristic can be measured by the angle at which the surface tilts when the water drops on the surface begin to roll down (Figure 7). The more hydrophobic the surface is, the larger the CA is, the smaller the angle of inclined surfaces is, and the smaller the area of contact between the liquid and the surface is, which reduces the temporal window and spatial possibilities for bio-adhesion events of bacteria from a contaminated droplet. When immersed in liquid, a liquid-air interface is formed between the hydrophobic surface and the liquid as a protective layer, which is difficult for bacteria to penetrate, thus inhibiting microorganisms from settling and adhering.

Silicon based materials have good hydrophobicity and are widely used in dental materials. The silicification of titanium implants is one of the most common applications. Using silane primer to siliconize the surface of titanium can significantly reduce the surface energy and improve hydrophobicity. Previous studies demonstrated that the preparation of silane primer using 3-acryloxypropyltrimethoysilane + bis-1,2-(triethoxysilyl)ethane increased the contact angle of the titanium surface and hydrophobicity and decreased the surface free energy, without affecting the surface roughness (Matinlinna et al., 2013). It was subsequently demonstrated that these changes in properties reduced the formation of *Candida albicans* colonies (Villard et al., 2015).

Besides titanium, introducing silicon based materials into the synthetic process of resin can give it an anti-adhesive property. Yu et al. synthesized a composite resin containing branched silicone methacrylate (BSM) (Yu et al., 2020; Tong et al., 2021). BSM was synthesized through a reaction between branched amino silicone and isocyanatoethyl methacrylate, and it was incorporated into 2,2-bis [4- (2-hydroxy-3-methacryloxy-propoxy) phenyl] propane (Bis-GMA)/triethyleneglycol dimethacrylate (TEGDMA) (50 wt%/50 wt%) with a series of concentrations to form resin matrices. The experimental composites (EC) were then prepared by mixing different resin matrices with silane BaAlSiO2 fillers. BSM can reduce the volume shrinkage of the composite resin. Adding 10 wt% or more BSM into ECs can make the CA of ECs >120°. With the BSM content increasing, the CA is larger. The addition of 15 wt% or 20 wt% of BSM gives ECs adhesion resistance to *Streptococcus pyogenes* without affecting the mechanical properties, but 30 wt% of BSM reduces the flexural strength of the resin material.

For orthodontic archwires, silicon treatment can also reduce bacterial adhesion. Inspired by the superhydrophobic antifouling principle of lotus leaves, Liu *et al.* (Tong et al., 2021) electrochemically etch orthodontic archwires (AWs) to improve the roughness of orthodontic archwires, after which 1H, 1H, 2H, 2H-perfluorodecyltrimethoxysilane (FAS) was deposited on the prepared AWs in a decompression environment at 80°C overnight. The CA of the treated orthodontic archwires were all above 120° and even reached 150°. Super hydrophobicity caused air to be trapped on the surface, which significantly reduced the actual contact area between the rough AWs and the bacterial suspension. It not only improved the corrosion resistance of the archwires and reduced the release of Ni ions, but also reduced the adhesion of the *S. mutans*.

However, subsequent experiments showed that superhydrophobic surfaces constructed with 1H, 1H, 2H, 2H-perfluorooctyltriethoxysilane lead to the disappearance of the air layer on the surface when it is immersed in water for a long time. The phenomenon is observed for a variety of superhydrophobic surfaces, suggesting that the anti-fouling of surfaces created by chemical modification is not durable (Hwang et al., 2018).

## Bioinspired slippery surfaces

In terms of stain prevention, pitcher plants are slightly different from fish scales and lotus leaves. The surface of pitcher plants is micro/nanotextures that lock in special liquids and build a slippery surface to resist stains. From pitcher plants, researchers design slippery liquid-infused porous surfaces (SLIPS), whose low-surface-energy porous solids are infiltrated by lubricating liquids to form a stable, immobilized, and smooth liquid-like omniphobic surface. Immiscible liquids deposited on the SLIPS can be easily removed even under weak shear forces, thus providing great promising for the resistance of fouling organisms (Zhang et al., 2017). The anti-fouling of hydrophobic interfaces created by SLIPS are stronger and more durable than those created by chemical modifications (Epstein et al., 2012; Howell et al., 2014; Amini et al., 2017).

**FIGURE 8:**
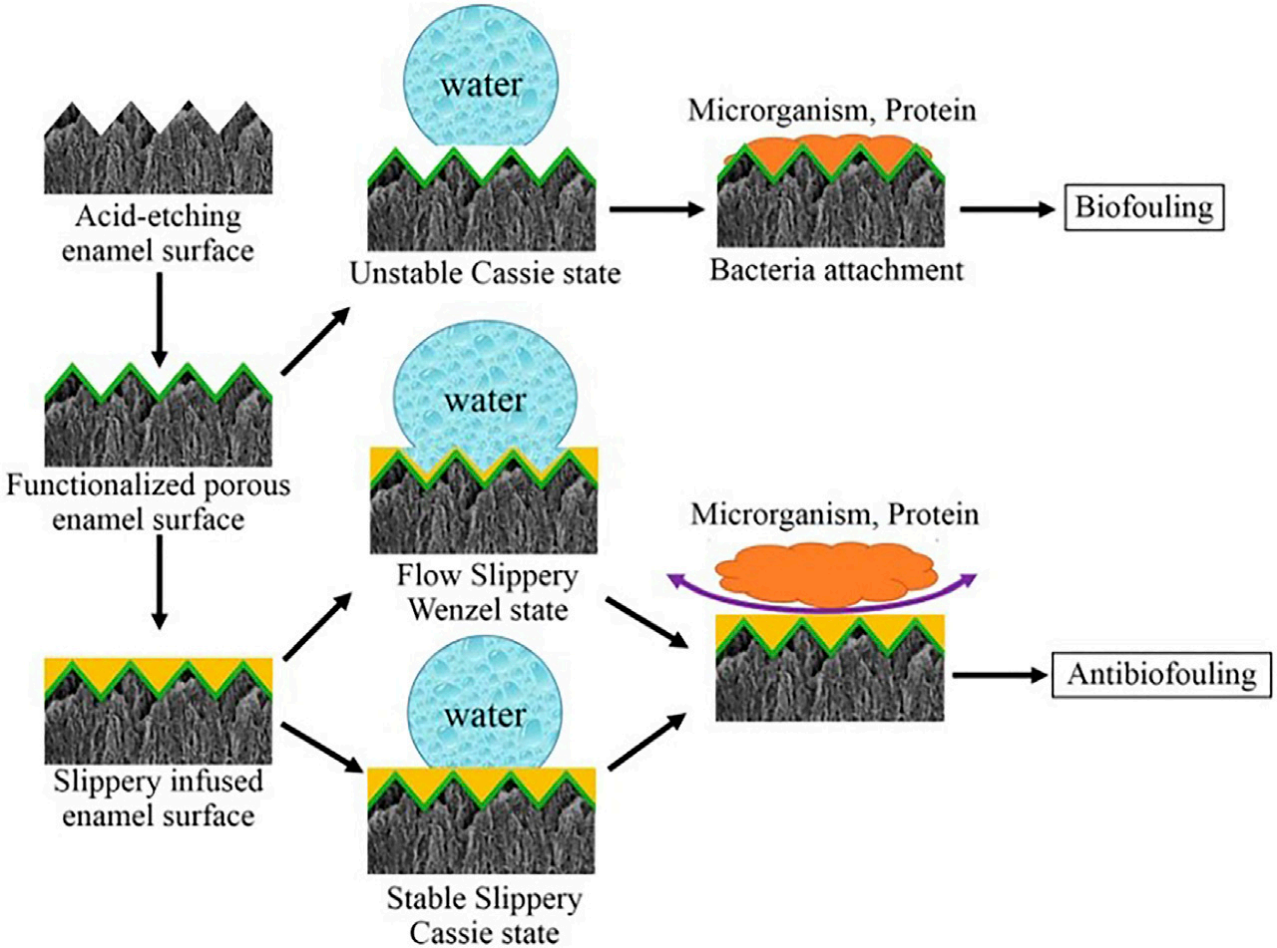
The process of anti-fouling on SLIPS. Copyright from ref (Yin et al., 2016).

Three important criteria for the design of a stable SLIPS are as follows: 1) the surface prefers to be rough to increase the adhesion of the lubricant and its immobilized surface area; 2) the chemical affinity between the lubricant and the solid should be higher than that between the surrounding fluid and the solid; 3) the lubricant and the surrounding fluid must be largely incompatible (Epstein et al., 2012). Based on the above criteria, Yin *et al.* (Yin et al., 2016) synthesized a SLIPS. Firstly, they use 37% phosphoric acid to etch enamel surfaces to obtain micro/nanoporous surfaces. Then, the surface is functionalized by hydrophobic low-surface energy heptadecafluoro-1,1,2,2-tetra- hydrodecyltrichlorosilane. Subsequent infusion of fluorocarbon lubricants (Fluorinert FC-70) into the polyfluoroalkyl-silanized rough surface results in an enamel surface with the slippery liquid-infused porous surface (SLIPS). The hydrophobic surface has been demonstrated to have an excellent anti-adhesive effect on *S. mutans in vivo* and in rabbits’ oral. The mechanism is that functionalized porous enamel surface is slightly hydrophobic which is easy for bacteria to adhere, while the lippery infused enamel surface has two states and both states is so super hydrophobic that microorganism and proteins are hard to adhere (Figure 8). And then, during simple dipping process, researchers used crystal violets to produce a crystal violet-impregnated slippery so that SLIPS has bactericidal feature (Patir et al., 2021).

SLIPS, as a new anti-fouling method, has few application in the oral field, and the corresponding *in vivo* and *in vitro* experiments need to be enriched, especially the stability in the oral cavity under special conditions.

## Conclusion

Inspired by biological anti-fouling phenomena in nature, super hydrophilic, super hydrophobic, and smooth surfaces have been successfully applied in dentistry to resist bacteria effectively. And preventing bacterial adhesion is achieved by an energy barrier or a trapped layer of water/air, that is, difficult for bacteria to penetrate through. However, super wettability dental materials still face the following problems: firstly, materials applied in human body need to be biocompatibility. Secondly, mechanical properties of materials may change when various components are mixed together, so more researches are needed to achieve anti-adhesion without reducing the mechanical properties of materials, or even improving them. Finally, the oral cavity is in a constant temperature and humidity environment, stability and durability of super wettable materials under such condition also need to be futher investigated. Most of the existing experiments are *in vitro* or *in vivo* in animal. Whether it is harmless to human need to be further explored. Super wettability materials with their unique physicochemical anti-adhesion mechanisms will become an increasing area for oral antimicrobial practice and provide a new direction for solving drug resistance.
